# Use of RWE to Inform Regulatory, Public Health Policy, and Intervention Priorities for the Developing World

**DOI:** 10.1002/cpt.2449

**Published:** 2021-10-31

**Authors:** Douglas McNair, Murray Lumpkin, Steven Kern, Daniel Hartman

**Affiliations:** ^1^ Global Health, Integrated Development Bill & Melinda Gates Foundation Seattle Washington USA

## Abstract

For low‐ and middle‐income countries (LMICs) to benefit from real‐world evidence (RWE)/real‐world data (RWD) in both product registration (“regulatory”) decision making and in product utilization policy (“policy”) decision making, they need to overcome several challenges. They need to deploy more electronic health records systems (EHRs), adjust for confounder variables, build trust between stakeholders, and create laws and regulations for local generation of data that are assented for secondary use. The role of procurers and their use of RWE/RWD in the LMIC context likewise is in a state of ongoing development. Procurers of health products are strong players currently in the “access” chain as LMICs continue to work on strengthening governmental health technology assessment (HTA) bodies. Procurers’ use of RWE is presently at an early stage and is mostly indirect, leveraging RWE results that are produced by researchers in high‐income countries (HICs), often under considerably different regulatory and policy objectives and constraints compared to LMICs’ epidemiology and priorities. Pending wider deployment of EHRs and other RWE sources, stakeholders must realize that populations from HIC RWE (i) can be devised to closely resemble phenotypic patterns in LMIC populations and (ii) can be analyzed to align with LMICs’ unmet needs.

## CURRENT STATE

Randomized controlled trials (RCTs) are the traditional gold standard for determining the safety and efficacy of medical products for regulatory decision making and for determining the safety and effectiveness of medical products for policy decision making, but the limited projectability, expense, and time to conduct RCTs have prompted many to look for other sources of reliable, interpretable data for robust evidence‐based decision making. This paper explores the use of RWE to support both regulatory decision making and policy decision making, including its use to help plan RCTs for both purposes and to provide reliable, interpretable data for evidence generation, which is complementary to that of RCTs. RCTs’ results quantitatively compare interventions to inform healthcare decision making. However, more comparisons are desired than can be conducted given constraints on time and funding, and point‐in‐time results from a completed RCT typically are not updated or extended to new populations. Stakeholders in LMICs and HICs alike are increasingly turning to RWE to inform their decisions, alongside evidence from RCTs.

RWE refers to the evidence produced by analyzing and/or synthesizing RWD. Data are raw materials that are noninformative by themselves. By contrast, evidence is produced from the data by statistical and machine‐learning analyses and denotes the interpretation of the information to instruct a conclusion or decision that may guide regulatory and/or policy decision making, order priorities, or inform practice. RWE is generated by analyzing data collected during routine care. RWE can address a variety of topics, including disease epidemiology, treatment efficacy, effectiveness and safety, and health economic value and impact.^1^, ^2^, ^3^ Over the past decade, applications of RWD and the derivative evidentiary works (RWE) produced from RWD have rapidly expanded from informing healthcare decisions at the patient and health network level to influencing health product decisions, including regulatory approvals and policy decisions regarding coverage.^4^ Comparative effectiveness research using RWE can enhance projectability for decision making; however, the lack of randomization means that biases and confounding require careful attention. Moreover, RWD are seldom defined by all stakeholders in an official, ratified specification.^5^ Some standards and best practices for assuring quality are in place, but other processes are either specific to the research question and RWD available or are yet ill‐defined.

The 21st Century Cures Act and Prescription Drug User Fee Act (PDUFA) recommend RWE as useful to supplement RCTs’ evidence on the efficacy, effectiveness, safety, and value of medical products in routine care. Similar recommendations have recently been promulgated by the European Medicines Agency (EMA) and other agencies. Four requirements have been proposed to enable successful decision making based on healthcare database analyses (meaningful valid expedited transparent (MVET))^6^: meaningful evidence that provides relevant and context‐informed evidence sufficient for interpretation, drawing conclusions, and making decisions; valid evidence that meets scientific and technical quality standards to allow causal interpretations to be derived; expedited evidence that provides timely incremental evidence that aligns with the regulatory and policy decision making process; and transparent evidence that is findable, accessible, interoperable, reusable, auditable, and reproducible, and therefore trustworthy for decision makers. RWE systems and processes that satisfy MVET requirements to a high degree increasingly contribute to regulatory decision making.^7^

RWE from EHRs and registries can occasionally serve as substitutes for RCTs. However, concerns about the validity of analyses of uncontrolled, nonrandomized data persist. Understanding how to implement a valid RWD analysis is prerequisite to relying on RWE in regulatory and policy decision making. By way of example, the Randomized, Controlled Trials Duplicated Using Prospective Longitudinal Insurance Claims: Applying Techniques of Epidemiology initiative (RCT‐DUPLICATE) implements a structured process to design RWE studies that emulate RCTs.^8^, ^9^, ^10^ Careful emulation of RCT inclusion‐exclusion criteria and end point definitions in RWD‐based studies is essential for reproducibility, as is selection of active comparator therapies with clinical indications and use patterns similar to those in an RCT.

In general, RWD repositories have minute‐wise time precision in representing when specific procedures and tests were performed, when clinical observations were made, when medications were dispensed, and so on. Such date‐time coordinates allow to utilize implementation of science methods that are oriented to ascertaining causal relationships, such as regression discontinuity analysis, structural equations modeling, Bayesian networks, and contingency analysis. Linkage across databases for laboratory test results, claims, retail pharmacy dispensing, EHRs, registries, sensor‐enabled wearable devices, and social media has great potential not only for regulatory and policy purposes but also for clinicians and patients. Reliable RWE that leads to causal inferences can help to optimize *in vitro* diagnostic tests, as well as reduce the chance of overuse or underuse of these tests, while providing timely, accurate diagnoses to improve the care of patients. Despite this fact, beyond completing and communicating research findings based on RWE, there are difficulties in implementing changes in health policy, including communication gaps between stakeholders, problematic political processes, reluctance of some policy makers to utilize research findings, and resistance to change.^11^ Involving stakeholders early in designing the objectives of a research program and throughout the research period improves RWE utilization and implementation effectiveness.

One example of this in an LMIC setting is a recent study of hydroxyurea treatment of sickle cell disease in Malawi.^12^ Another recent example involves the clinical effectiveness of treating all persons living with HIV (PLWHs) in Zambia.^13^ Altering treatment eligibility may induce behavioral changes, such as continuity of care and loss‐to‐follow‐up or lead to unanticipated consequences (for example, depletion of limited local health services capacity, leading to underservice of sicker PLWHs). Mody *et al*.^13^ assessed the impact of changing Zambia’s HIV treatment guidelines liberalizing the threshold for treatment eligibility from 350 to 500 CD4 cells/μL. Using an RWE regression discontinuity design, they found that this change in policy was associated with a prompt rise in antiretroviral therapy (ART) initiation as well as enhanced retention among newly treatment‐eligible patients with no adverse impact on patients having lower CD4 levels. Mody *et al*. estimated that the change in guidelines led to a 37.9% increase in retention in care and the number needed to treat = 2.6 patients would need to be initiated on ART to prevent one incremental instance of loss‐to‐follow‐up in ART treatment. Under such a policy, expanding ART eligibility was associated with improvement in patient adherence and care continuity behaviors that were not observed in RCTs.

Distributive justice and equitability of health care require that interventions that are known to be effective be implemented at scale in a timely manner, including in resource‐poor settings, such as LMIC communities.^14^ Achieving this objective requires high‐quality implementation research and pragmatic studies that accommodate the complexities of real‐world contexts. Often, there is a need to determine whether existing evidence is sufficient to bridge findings from previous implementation research to a new setting, or whether additional RWE or a new RCT is needed.^7^ Brian Haynes’ seminal publication in 1999: “Can it work? Does it work? Is it worth it?” remains instructive for both the current state and the future of RWE in priority‐setting and regulatory and policy decisions.^15^ Therapeutic impact in real‐world communities depends not only on nominal efficacy but also on diagnostics’ performance, clinician compliance, patient adherence, and the coverage and processes of health services. Misdiagnosis can result in inappropriate overutilization or underutilization, or delays in people receiving appropriate treatment. The reality is that providers often fail to prescribe or administer the treatment according to labeling and established guidelines, and free‐living ambulatory patients often are nonadherent and take less than half of prescribed treatments.

RWE can help to assess market size and likely impact for the population in the catchment areas from which the RWD originated, given the contemplated labeling: how many real‐world patients in those geographies would be eligible for RCT inclusion by applying the RCT selection criteria to the prevalent real‐world populations; but this can be a chicken‐vs.‐egg proposition. If there are not sufficient RWD cases with exposure to a therapeutic, then policy‐makers may withhold coverage decisions. Public and private payers use concerns regarding real‐world therapeutic value as a basis to assert “unquantifiable benefit” at the time of market registration. Pharmacy and Therapeutics committees in turn delay placing new products on formulary, which limits product availability and distribution. All these present barriers to widespread real‐world utilization and accrual of RWD to demonstrate effectiveness and cost‐effectiveness. Such barriers are a particular problem in LMICs.

To have a durable impact, health policy decisions must be relevant, evidence‐based, and transparent.^16^ Decision‐analytic modeling supports the implementation process,^17^ but its role is reliant on its credibility. RWE is yet seldom cited in policy‐making materials, even in therapeutic class reviews where RWE is readily available.^18^, ^19^ In this connection, partnerships between researchers and policymakers may improve uptake and integration of scientific evidence. Zaniewski *et al*.^20^ describe the research‐policy partnership between the International Epidemiology Databases to Evaluate AIDS (IeDEA; https://www.iedea.org) and the World Health Organization (WHO), which was established in 2014. IeDEA is an international research consortium, which analyses data on ~ 2 million PLWHs under care in real‐world settings in 46 countries in Asia‐Pacific, the Caribbean, Central and South America, North America, and sub‐Saharan Africa. To date, the partnership has been successful: RWE‐informed discussion of WHO policy agendas has led to more policy‐framed and timely research, and the collaboration has provided the WHO with prompt access to continually updated RWE regarding effectiveness and safety.

## FUTURE STATE

Increasingly, health policy decision makers in LMICs aim to utilize RWE to inform health systems planning, costing, policy, and implementation, as well as to accelerate and improve probability of success of RCT designs.^21^ Yet, there is still much that remains unknown about (i) the types of evidence that are most convincing for LMIC policymakers and community groups, (ii) the factors that facilitate or impede the decision‐making process, and (iii) the difficulties that arise when implementing research results in care processes in low‐resource contexts. Policy and policy processes are often fiercely contested, involve multiple actors with different concerns, priorities, and values, and are influenced by a range of contextual factors.

A responsibility of regulators and policy makers is to determine that evidence indicates a favorable benefit‐risk profile for the affected population throughout the product lifecycle.^22^, ^23^ An important step in this process is to render registration and policy decisions that interdict substandard, unsafe, ineffective, or cost‐inefficacious therapeutics from entering the market. Decision‐makers might ideally wish to have overwhelmingly convincing evidence, but there is a balance to be considered: demands for extremely strong evidence will delay the provisioning of good products and increase their cost, and, inversely, reliance upon insufficiently strong evidence will allow some inferior or bad products to slip through into general use, consuming resources, and producing harm or yielding little benefit. In the case of the regulatory marketing approval process, a proportion of therapeutics that do not have an adequate, intended clinical effect will be granted licenses—a decisional false‐positive rate—and a proportion of therapeutics that are effective will be rejected—a decisional false‐negative rate. In real‐world contexts of implementation research based on RWD, one faces the same issue of maintaining an adequate strength of evidence, keeping the same false‐positive rate and false‐negative rate no matter what form of evidence is used. The preparedness to tolerate false‐negative errors may be diminished in LMIC settings where therapeutic options and supplies are limited, compared to HIC contexts.

There are significant remaining challenges with RWE, such as heterogeneous perspectives and differences in outcome measures in RWE generation^24^ and these challenges are more pronounced in diverse LMIC contexts compared to typical HIC contexts. However, RWE is clearly useful in HTA policy making,^25^ and this is anticipated to be increasingly so for LMICs. As a point of reference, since 2017, the EMA has offered consultations in parallel with the European Network for Health Technology Assessment (EUnetHTA), enabling access to and feedback from regulators and HTA bodies on sponsors’ evidence‐generation plans to support decision making on marketing authorization and financing of new medicines at the same time. The procedure is a unified mechanism for sponsors to jointly consult with EMA, EUnetHTA, and HTA organizations on evidence‐generation plans. This may be a good model for the African Medicines Agency (AMA) and other agencies to adopt or adapt.

In the years ahead, there is a need to develop hybrid study methodology combining the best parts of RCTs and observational RWE study designs to produce combined evidence that enables timely, reliable regulatory and policy decision making, public communications, and social marketing.^26^ The recent experience with coronavirus disease 2019 (COVID‐19) therapeutics and vaccines has afforded a prime example of how an early paucity of evidence and the lack of an adequate communication plan can attenuate RWE uptake and delay or obviate the hoped for impact of implementation. Pragmatic trials, including RWE with recent historical controls, led to regulatory approval of avelumab, blinatumomab, and paliperidone palmitate, after the United States, the 21st Century Cures Act, and PDUFA VI. Similar RWE‐oriented acceleration of drug development is important for LMICs and the regulatory agencies serving those countries.

Finally, the use of EHRs, administrative data, and other RWD datasets to evaluate health care technologies and programs has expanded over the past decade, especially in HICs, but to date LMIC RWD lags, not only in systems deployment but also in regulations and policies governing secondary use of de‐identified data for observational research. To date, RWE has been used predominantly to perform postmarketing surveillance to monitor drug safety and detect adverse events.^27^, ^28^ RWE has also been effective in situations involving chronic and subchronic end points or when performing RCTs is problematic, such as in neonatology or obstetrics. Strengths, limitations, and recommendations for RWE utilization appear in **Table** **1**.

**Table 1:** RWE profile – strengths, weaknesses, recommendations

Strengths of RWE
Great diversity in inclusion and exclusion criteria, providing information on treatments in patient groups that are usually excluded from RCTs Reflects the actual clinical and logistical and financial aspects of implementing the treatment Reflects the local culture, values, priorities, and practices of citizens in policy‐relevant catchment areas, improving local participation, and social ownership of policy‐making Large samples are advantageous for active pharmacovigilance studies of uncommon adverse drug reactions and adverse events that require long time to materialize RWE can have very large sample sizes, enabling discovery of new biomarkers relevant to treatment decision making, policy and health care finance, and analysis of subgroups Quicker and far less expensive than RCTs and can be repeated ad lib to monitor changes over time and safety and effectiveness in different locales Can support rapid responses to unanticipated and emergent situations Can support model‐informed drug development and anticipative Target Policy Profile decisions relative to standard‐of‐care Can support analyses of longitudinal processes and high‐dimensionality problems whose mechanisms of causation and treatment may be unclear Can support implementation science and statistical methods to ascertain causal relationships and sequence association rules Can assess a broad range of outcomes, far beyond what is practical in RCTs
Weaknesses of RWE
Inability to evaluate investigational products prior to regulatory approval Risk for bias unless addressed by propensity score adjustment, randomization, etc. Limited ability to assess maternal‐neonatal or other outcomes that involve record‐linkage between family members (constrained by applicable privacy law and regulation) Limited ability to assess outcomes that occur in ambulatory/home settings or that are associated with social stigma Limited ability to assess interaction of tobacco, vaping, alcohol, illicit drug use, or incarceration with treatment regimen outcomes Limited ability to assess interaction of socioeconomic variables and social determinants of health with treatment regimen outcomes (constrained by applicable privacy law and regulation) Limited ability to assess psychiatric treatment regimens and outcomes (access to unstructured clinical narrative text constrained by applicable privacy law and regulation) Only provide a robust basis for comparing treatment regimens and treatment intensities and durations that are relatively common in current practice A patient may request or decline specific treatments based on advertising or her own research, such that clinicians’ therapeutic decisions may be affected or obscured in unknowable ways Initially randomized subjects included in RWD‐based study experience changes in treatment over time, necessitating censoring from final cohort for analysis Data sources have different objectives and are subject to specific limitations with respect to the disease and therapy‐relevant analytical options Extracted electronic medical data records can have severe between‐site heterogeneity Variable frequency and duration of exams and measurements, depending on insurance coverages and clinician decision‐making behaviors Large amount of missing data and loss‐to‐follow‐up, depending on insurance coverages and clinician decision‐making behaviors It is difficult to confirm whether the drug was taken appropriately, except with Medication Administration Records in acute care settings Diagnosis can be unreliable and susceptible to both patient and clinician biases, especially for those based upon clinical symptoms only
Recommendations for RWE
Increase deployment of EHR systems in LMICs Improve policies and systems for record‐linkage, secondary‐use, and data rights for multilateral provisioning of RWD for Helsinki Committee‐approved observational research in LMICs Establish privacy law and regulations in LMICs governing ethical re‐use of de‐identified RWD for secondary purposes in public health and observational research Integrate diverse sources of RWD (including waveform and high‐frequency data from sensor‐enabled wearable devices and patient‐reported Medication Administration Records and outcomes data via mobile devices apps) to improve the scope of RWE in certain conditions that have infrequent assessments by clinicians Standardize RWE data model, ontologies cross‐walks, data collection, processing, quality assurance, archival, recovery, and auditing Unify RWE quality and heterogeneity standards Agree on methods that produce and verify high‐quality RWE

EHR, electronic health record; LMICs, low‐ and middle‐income countries; RCTs, randomized controlled trials; RWE, real‐world evidence.

It is by now clear that RWE can inform decisions on how best to use available and emerging health care technologies. We emphasize that in so doing RWE does not “substitute for” RCT evidence so much as (i) accelerate the product development process in a manner that is aligned with real‐world realities, and (ii) de‐risk development strategies and RCT study designs. In that regard, RWE serves as a sort of “parallel” or corroborative evidence that complements or supplements prospective RCT evidence. In some instances, especially where event rates are low (e.g., newly incident HIV cases in the pre‐exposure prophylaxis era) and/or irreversible (e.g., neonatal or maternal mortality), RWE can help to ensure that RCT study designs provide the best chance for benefit and the lowest risk to human research subjects. These aspects are always important but are particularly so in situations that involve low‐resource care settings, populations who have difficulty traveling to obtain diagnostic or care services, populations who are vulnerable or who experience ethnic discrimination, and the like. In yet other instances, RWE can help to determine whether equipoise is present, which is essential for the selection of a control arm, for informed consent, for ethical stopping for efficacy or futility, and other aspects. In terms of HTA and policy decisions, it is vital that a new therapeutic or regimen yield a minimum clinically important difference compared to standard‐of‐care (SoC) therapies. In still further instances, RWE can establish the incremental cost‐effectiveness ratio (ICER) for SoC predicate therapeutics, which then can serve to guide setting the target policy profile for the development program. That is, for HTA and policy‐making, superiority of the test article in terms of dollars‐per‐disability‐adjusted life year and ICER is preferred, but ICER noninferiority is essential for sound policy.

Given the frequent misunderstandings of what can and cannot be done with RWE, we note that it is not only possible to emulate or replicate RCTs in contemporaneous RWD with end points and selection criteria that closely match those of the contemplated RCT; it is also possible with longitudinally linked EHR derived General Data Protection Regulation (GDPR)‐ and Health Insurance Portability and Accountability Act (HIPAA)‐compliant RWD to conduct prospective studies in the RWD repositories using randomization processes applied to the prevalent patients whose longitudinal patterns of care and outcomes are captured in the institutions sourcing the RWD. Longitudinal record‐linkage in contemporary RWD repositories that continuously accrue data can enable randomization of subjects from an index date, closely replicating what would otherwise have been done in an RCT. Ordinarily, generation of RWE does not involve random assignment of subjects to treatments, and therefore advanced propensity score matching and statistical techniques are needed to control for selection biases, immortal time bias and lead‐time bias, and especially confounding by indication and severity. Nevertheless, these state‐of‐the‐art approaches to control for bias are likely unfamiliar to healthcare decision makers. A lack of understanding may lead decision makers to mistrust and place a lower importance on information from such studies, limiting their use in the decision‐making process. Therefore, decision makers may instead rely excessively on familiar sources of evidence, such as RCTs, or use expert opinion.

**Figure 1:**
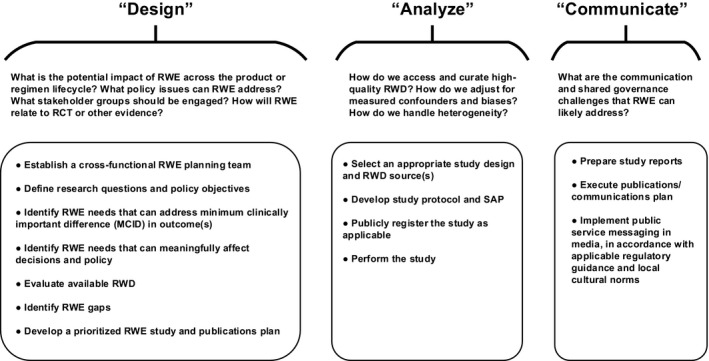
Modes of RWD application in integrated development. RCT, randomized controlled trial; RWD, real‐world data; RWE, real‐world evidence.

As noted in **Figure** **1**, in the Design phase, RWE can help improve the specification of research questions that can be addressed in an RCT, prior to writing a research protocol for said RCT. Using RWE in this way ensures that investigators have identified the relevant decision criteria in the context of the low‐resource population and the strategic issues and priorities that must be addressed in that context. In the Analyze phase, the use of valid epidemiological approaches and propensity score methods can reduce confounding and biases arising from non‐randomized data.^29^ In the Communicate phase, RWE can facilitate establishing consensus among stakeholders and decision making regarding the value and local impact of a product or regimen in practical, real‐world contexts, such as those that are likely to prevail in populations in low‐resource geographies.

## DISCUSSION

According to the International Society for Pharmacoeconomics and Outcomes Research (ISPOR), RWD can be any data that goes beyond what is normally collected in traditional phase III clinical trial programs and is used for clinical, coverage, and payment decision making. As such, RWE derived from RWD now routinely contributes to postmarket REMS‐type evidence for policy making and revising. However, recently, RWE is finding new, valuable applications in model‐informed drug development and decision making that anticipates target policy profile to accompany integrated product development plans. In other words, policy‐making should not be an afterthought that is deferred until after RCTs are completed and regulatory registration is granted and RWD becomes widely available. Instead, the entire development program and investment decisions should anticipate what minimum effectiveness impact would be sufficient to justify changing policy from the perspective of current SoC and should design the RCTs and RWE studies accordingly.

At present, LMICs lag significantly in producing and using RWE in regulatory and policy decision making. The trend, however, is changing due to several factors that influence the future of the healthcare industry in these countries, including in‐country and regional pharmaceutical manufacturing and regulatory agencies as well as low‐cost EHRs systems, such as OpenMRS (https://openmrs.org/) and AMPATH (https://www.ampathkenya.org/research) that utilize a common data model, a standardized ontology and nomenclatures, and have the means for making available GDPR‐compliant de‐identified privacy‐protected individual‐level longitudinally linked detailed RWD. Evolving health challenges in LMICs, changing population demographics and epidemiology, increased emphasis on regulatory harmonization, increased attention to local clinical and economic alignment, sponsors’ incentivization by global trends in health care, financial return hurdles for product development, and other factors add to the complexity of financing and uptake of RWE in LMIC settings. For LMICs to achieve the benefits of RWE in healthcare strategy and policy, they need to address several challenges, including building trust between stakeholders, establishing reliable databases, and extraction and de‐identification processes used to produce RWD suitable for conducting high‐quality RWE studies while maintaining patients’ confidentiality.

For many clinical indications where the target condition and frequent comorbid conditions have significant prevalence in HICs, adequate relevant RWD is available to statistically power active pharmacovigilance analyses for safety signals and analyses of effectiveness end points.

Notwithstanding certain pharmacogenomics and ethnic variations in LMICs that may give rise to differences in effectiveness or safety, available HIC data is, in our experience, broadly consistent with signals and effect sizes that are later measured and verified in LMIC populations. However, when there is negligible prevalence of a condition or a constellation of comorbid conditions and their treatments in HIC populations, then RWD from local LMIC populations is essential. For instance, even though the anti‐malarial tafenoquine and the anti‐mycobacterial bedaquiline received US Food and Drug Administration (FDA) approval in 2018 and 2012, respectively, there are as yet too few patients exposed to either of these drugs in the major commercially available HIC EHR‐derived RWD repositories to evaluate tafenoquine and bedaquiline regimens’ safety or efficacy. For such use cases, one awaits RWD from the LMICs’ local populations in whom such drugs are extensively prescribed. In other instances, such as moxidectin and other agents indicated for neglected tropical diseases, the pattern of prescribing the drug in HICs may differ substantially from prescribing patterns in LMICs. The stage and severity of disease at the time of presentation may not be comparable, the durations of treatment or repeated courses of therapy may differ between the geographies. The dosing and dose‐adjustment to account for concomitant medications and likely drug‐drug interactions that might arise with the target medication may differ. Comorbid conditions or other covariables may also be different, such that safety and efficacy end points ascertained in HICs’ RWD may depart significantly from those same end points ascertained in LMICs’ RWD. For example, onchocerciasis encountered in migrants or returning travelers in HICs is predominantly in adults and is generally of recent onset and unlikely to be associated with comorbid *Loa loa* infection. The risk of encephalopathy or severe edema or aggravation of onchodermatitis with moxidectin treatment of prevalent onchocerciasis in adult patients is distinctly lower in HICs’ RWD than the risk of these serious adverse events in patients in LMICs. By contrast, in LMICs, considerable prescribing of moxidectin occurs in pediatric patients, for whom available HIC onchocerciasis RWD offers little or no insight. Furthermore, the preferred dosage form and route of administration for certain drugs may differ on a country‐by‐country basis, influenced by procurement and logistical issues or other factors in LMICs. Thus, reasonable care must be taken to ensure the comparability of regimens and phenotypic features of patients in HICs and LMICs in order that the HIC RWD be relevant clinically and pharmacologically to LMIC populations.

In the case of new molecular entities having novel mechanisms of action, there may be no extant RWD for such compounds until weeks after their first marketing approval in HIC or LMIC jurisdictions. Nonetheless, RWD for outcomes that are associated with previously approved therapeutics can serve as SoC comparators to determine what would be deemed to be of value in LMIC locations or to determine what would constitute clinically relevant superiority of the new molecular entity in terms of efficacy or safety (or both) in any geography.

Beyond RWD to inform clinical trial design in LMICs and HICs, LMICs’ RWD is recently beginning to support active pharmacovigilance (PV). The need for this was established many years ago^30^ but implementation of PV systems and processes has depended on alignment of local LMIC national regulatory authorities (NRAs), epidemiology services (Centers for Disease Control and Prevention (CDCs)), and other stakeholders, which has been forthcoming only in the last several years. For example, the African Union NEPAD Smart Safety Surveillance (AU‐3S programme^31^, ^32^) has adapted HIC regulatory agencies’ principles to fit local needs and capabilities in sub‐Saharan African nations. During 2020 and 2021, Ghana, Nigeria, South Africa, and Ethiopia leveraged PV technology provisioned by the UK Medicines and Healthcare Products Regulatory Agency (MHRA)’s information technology unit and established an International Conference on Harmonization (ICH)‐compliant RWD data repository in a secure UK cloud environment. Production operation of this PV system commenced in June 2021. More progress for LMICs’ RWD and PV safety signal detection is anticipated in the near future. With regard to the role of payors in the use and interpretation of RWE, one might further anticipate that local LMICs’ RWD on adherence for products or regimens of comparable complexity, therapeutic index, cold‐chain requirements, and the like may serve to demonstrate adequate logistical capacity for importation and safe and effective use of a new product that has attributes similar to said products and regimens. In low‐income countries, products may be procured by third parties rather than by the Ministry of Health. For example, the AU‐3S program noted above has during 2021 pooled African COVID‐19 vaccine safety data for vaccines from the 4 currently participating African countries, enabling vaccine safety surveillance and decision‐making for 4 COVID vaccines by those countries’ NRAs.

Pending the wider deployment of RWD‐capable EHRs, claims systems, and other RWD sources in LMICs, pharmaceutical firms, regulators, and agencies, such as the WHO, should recognize that populations from HIC RWE can be devised in such a way as to closely resemble phenotypic patterns present in LMIC populations. Due to travel, immigration, and location where care services were eventually delivered, even neglected tropical diseases and other low‐prevalence conditions do have cohorts of considerable size in some HIC‐based RWD repositories. These non‐local RWD cohorts can be useful for estimating effect‐sizes that are likely to prevail in LMIC locations, for establishing rational selection criteria for prospective RCT designs, for developing novel biomarkers for adaptive trials, for provisionally optimizing end points design, and for performing clinical trial simulations in advance of conducting clinical trials in the LMIC locations where local EHRs and RWD are presently lacking. Apart from the Bill & Melinda Gates Foundation’s funding research that leverages de‐identified in‐country RWD from local EHR systems like OpenMRS and AMPATH, we make extensive use of HIC‐based RWD applying LMIC‐like phenotype pattern filters to emulate and cross‐validate LMIC RWE. In short, a tremendous amount of work lies ahead for increasing the use of RWE for regulatory and policy decision‐making purposes for low‐income countries and for optimizing the implementation of diagnostic and therapeutic regimens in daily practice. Nonetheless, the future holds great promise and merits collaborative investments by all stakeholders. Our perspective is that evidentiary quality^33^ is fundamental to the impact that RWE can have on regulatory and policy decision quality, not only in geographies where RWE is already established, but also in new areas such as LMICs.

## FUNDING

No funding was received for this work.

## CONFLICT OF INTEREST

All authors declared no competing interests for this work.
